# Association between high preoperative body mass index and mortality after cancer surgery

**DOI:** 10.1371/journal.pone.0270460

**Published:** 2022-07-08

**Authors:** Jungchan Park, Seung-Hwa Lee, Jong-Hwan Lee, Jeong Jin Min, Ah Ran Oh, Kyunga Kim, Joonghyun Ahn

**Affiliations:** 1 Department of Anesthesiology and Pain Medicine, Samsung Medical Center, Sungkyunkwan University School of Medicine, Seoul, Korea; 2 Division of Cardiology, Department of Medicine, Heart Vascular Stroke Institute, Samsung Medical Center, Sungkyunkwan University School of Medicine, Seoul, Korea; 3 Statistics and Data Center, Research Institute for Future Medicine, Samsung Medical Center, Seoul, Korea; 4 Department of Digital Health, SAIHST, Sungkyunkwan University, Seoul, Korea; University of Brescia: Universita degli Studi di Brescia, ITALY

## Abstract

Despite an association between obesity and increased mortality in the general population, obesity has been paradoxically reported with improved mortality of surgery and some types of cancer. However, this has not been fully investigated in patients undergoing cancer surgery. Using a cohort consisting of mostly Asian population, we enrolled 87,567 adult patients who underwent cancer surgery from March 2010 to December 2019. They were divided into three groups according to body mass index (BMI): 53,980 (61.6%) in the normal (18.5–25 kg/m^2^), 2,787 (3.2%) in the low BMI (<18.5 kg/m^2^), and 30,800 (35.2%) in the high BMI (≥25 kg/m^2^) groups. The high BMI group was further stratified into overweight (25–30 kg/m^2^) and obese (≥30 kg/m^2^) groups. The primary outcome was mortality during three years after surgery. Following adjustment by inverse probability weighting, mortality during three years after surgery was significantly lower in the high BMI group than the normal (4.8% vs. 7.0%; hazard ratio [HR], 0.69; confidence interval [CI], 0.64–0.77; p < 0.001) and low BMI (4.8% vs. 13.0%; HR: 0.38; CI: 0.35–0.42; p < 0.001) groups. The mortalities of the overweight and obese groups were lower than that of the normal group (7.0% vs. 5.0%; HR: 0.72; CI: 0.67–0.77; p < 0.001 and 7.0% vs. 3.3%; HR: 0.57; CI: 0.50–0.65; p < 0.001, respectively). This association was not observed in female patients and those undergoing surgery for breast and gynecological cancers. High BMI may be associated with decreased mortality after cancer surgery. Further investigations are needed for clinical application of our finding.

## Introduction

Obesity has emerged as a major health issue in the modern era. Obesity-induced in insulin metabolism and sex hormones, activation of growth factor signaling, induction of specific lipids, and secretion of various adipokines and inflammatory cytokines such as tumor necrosis factor-α, and interleukin-6 are associated with an increased risk of mortality [1,2]. There is convincing evidence for a biologic link between obesity and increased risk for the development of cancer as well as for metabolic syndrome [3,4]. However, some epidemiologic studies have reported that obesity may protect some types of cancer and reduce incidence and mortality [5–8]. This hypothesis that obese patients may have a better prognosis, generally referred to as the “obesity paradox”, has been relatively well demonstrated in critically-ill patients such as those with coronary artery disease, chronic pulmonary obstructive disease, acquired immune deficiency syndrome, chronic renal failure and those undergoing major surgeries [9–11].

Surgical resection is an established treatment modality for solid tumor [12], and more than 80% of cancer patients, estimated to be nearly 10 million cases every year need surgery for cancer [13]. Previous studies reported the obesity paradox for particular types of cancer surgeries [14–17], but the association between BMI and postoperative outcomes has not been fully investigated thoroughly in all types of cancer surgeries. In this study, we used the data from 10 years of consecutive cancer surgeries performed at a single large-volume tertiary center and evaluated the hypothesis that preoperative BMI is associated with mortality after cancer surgery. In addition, we evaluated the association according to cancer site to provide clinical perspectives.

## Materials and methods

### Ethics approval

Ethical approval for this study (SMC 2020-04-027) was provided the Institutional Review Board of Samsung Medical Center, Seoul, Korea (Chairperson Prof OJ. Kwon) on 8 April 2020. The Institutional Review Board of Samsung Medical Center waived the need for individual written informed consent, because the entire dataset was initially extracted in de-identified form. The cohort for this study was registered at https://cris.nih.go.kr (KCT0005000) before patient enrollment. This study was conducted in compliance with the declaration of Helsinki and was reported according to the Strengthening the Reporting of Observational studies in Epidemiology guideline.

### Study population and data curation

This observational cohort study used data from the Samsung Medical Center Cancer Surgery (SMC-CanSur) registry. This registry is a de-identified cohort consisting of 87,621 consecutive adult patients who underwent cancer surgery, defined as surgical removal of a solid tumor and adjacent tissue at any site including the brain, neck, breast, thorax, abdomen, colon, and pelvis from March 2010 to December 2019 at Samsung Medical Center, Seoul, Korea. After excluding patients without a preoperative BMI record from the registry, patients were stratified into normal, low BMI, and high BMI groups according to preoperative BMI, and the mortalities were compared in a pairwise fashion. The high BMI group was further divided into overweight and obesity groups, and mortalities in these groups were compared with that of the normal group in a pairwise fashion.

All SMC-CanSur registry data were extracted using the “Clinical Data Warehouse Darwin-C” of Samsung Medical Center. This is an electronic system built for investigators to search and retrieve de-identified medical records from the institutional electronic archive system, which contains records of more than 2.2 million surgeries, one billion laboratory findings, 100 million disease codes, and 200 million prescriptions. When cancer patients are registered in this system, all medical data relevant to cancer such as diagnosis date, cancer stage, metastasis, cancer treatment, and recurrence are separately organized and updated. Mortality data in this system are consistently validated with and updated according to the National Population Registry of the Korea National Statistical Office using a unique personal identification number for mortality statistics at institutions other than ours.

### Definitions

The high BMI was defined as a preoperative BMI equal to or greater than 25 kg/m^2^, and a BMI of less than 18.5 kg/m^2^ was defined as low BMI. Patients with a BMI equal to or greater than 30 kg/m^2^ in the high BMI group were further divided as obese patients according to the Centers for Disease Control and Prevention (CDC) guidelines. Previous medical history and underlying disease of patients were organized by integrating International Classification of Disease code-10 administrative data and manual review of preoperative evaluation sheet [18].

### Study endpoints

The primary study endpoint was all-cause mortality during three years after surgery. As secondary endpoints, five-year mortality, overall mortality, mortalities within the first year and 30 days, and recurrence rate were assessed.

### Statistical analysis

Categorical variables are presented as numbers with incidence, and continuous variables are presented as mean ± standard deviation (SD) or medians with interquartile range (IQRs). Baseline characteristics were compared by analysis of variance or the Kruskal-Wallis test. We compared incidences of outcome between the three groups in a pairwise fashion.

To maximize the study power by balancing confounding variables between the three study groups, we generated weighted Cox proportional-hazards regression models with adjustment by inverse probability weighting (IPW) using the ‘twang’ package in R programming [19]. Our statistical adjustment retained all available confounding factors including demographic data, comorbidities, preoperative treatment for cancer, and operative variables. Inverse probability weights were defined as the reciprocal of propensity scores, calculated by the generalized boosted model to estimate the average treatment effects. Generalized boosted regression was used to estimate the probability of being in each of the three study groups instead of a multinomial logistic regression model. Variables retained in the adjustment included age, male, smoking, alcohol, underlying diseases, preoperative care, and operative variables. For comparison between the three groups, we maintained the alpha level at 0.05 for the primary outcome by adopting a post-hoc Bonferroni’s correction, with a significance criterion of 0.0167 (0.05/3). The risks of mortality are reported as hazard ratio (HR) with 98.3% confidence intervals (CIs). Kaplan–Meier estimates were used to construct survival curves of the three groups, and these were compared using the log-rank test. We also constructed smoothing HR plots according to preoperative BMI. For sensitivity analysis, the observed association was evaluated in female patients, patients older than 60 years old, and patients without hypertension and coronary artery disease, use of metformin, use of statin, and metastasis. To reveal the effect on our result and minimize the possibility of bias from different cancers, subgroup analyses were performed according to cancer sites, and these results are presented in forest plots. All statistical analyses were performed with R version 4.0.2 (R Foundation for Statistical Computing, Vienna, Austria).

## Results

### Patient characteristics

After excluding 54 patients without a recorded preoperative BMI, a total of 87,567 adult patients undergoing cancer surgery were divided into the three groups according to BMI as follows: 53,980 (61.6%) were in the normal, 2,787 (3.2%) in the low BMI, and 30,800 (35.2%) in the high BMI groups. Baseline characteristics are summarized in Table 1. The median durations between cancer diagnosis and surgery were 34, 32, and 35 days in the normal, low BMI, and high BMI groups, respectively. Patients in the high BMI group tended to be older and male with higher incidences of smoking and chronic lung disease. Incidences of preoperative metastasis and anemia were lower in the high BMI group than the other two groups. After adjustment with IPW, absolute standardized mean differences among three groups were lower than 0.1 for all variables, suggesting that confounding variables well-balanced among the three groups.

**Table 1:** Baseline characteristics according to preoperative body mass index.

	Normal (n *=* 53980)	Low BMI (n *=* 2787)	High BMI (n *=* 30800)	Before IPW ASD	After IPW ASD
BMI	22.3 (±1.7)	17.5 (±0.9)	27.5 (±2.3)		
Age, years	55.4 (±12.9)	52.5 (±16.2)	57.0 (±12.1)	21.3	3
Male	22717 (42.1)	958 (34.4)	16353 (53.1)	25.5	6.5
Current smoking	16065 (29.8)	763 (27.4)	10986 (35.7)	11.9	1.5
Hypertension	13814 (25.6)	436 (15.6)	13500 (43.8)	42.9	5
Diabetes	10181 (18.9)	489 (17.5)	7506 (24.4)	11.2	1.7
Preoperative metastasis	1839 (3.4)	152 (5.5)	766 (2.5)	10.2	1.2
Coronary artery disease	1487 (2.8)	57 (2.0)	1311 (4.3)	8.5	1.9
Heart failure	84 (0.2)	7 (0.3)	66 (0.2)	1.4	1.8
Stroke	1102 (2.0)	56 (2.0)	772 (2.5)	2.2	3.3
Deep vein thrombosis	77 (0.1)	8 (0.3)	60 (0.2)	2.1	1.7
Peripheral arterial occlusive disease	67 (0.1)	4 (0.1)	33 (0.1)	0.7	1
Chronic kidney disease	404 (0.7)	21 (0.8)	322 (1.0)	2.1	2.2
Chronic lung disease	1636 (3.0)	141 (5.1)	815 (2.6)	8.4	0.4
Dementia	66 (0.1)	10 (0.4)	41 (0.1)	3.2	1
Chronic liver disease	3071 (5.7)	106 (3.8)	1786 (5.8)	6.2	6
Tuberculosis	2614 (4.8)	209 (7.5)	894 (2.9)	14	
Preoperative anemia	13479 (25.0)	1137 (40.8)	5255 (17.1)	36	2.1
Preoperative care					2.8
Chemo therapy	2363 (4.4)	190 (6.8)	1185 (3.8)	8.8	0.5
Radiation therapy	1540 (2.9)	127 (4.6)	806 (2.6)	7	1.2
Hormone therapy	130 (0.2)	7 (0.3)	80 (0.3)	0.3	0.6
Intensive care unit	92 (0.2)	5 (0.2)	53 (0.2)	0.1	
Continuous renal replacement therapy	2 (0.0)	0	0	0.6	1
Operative variables					0.5
Operation duration, minutes	166.3 (±94.8)	169.4 (±103.5)	173.4 (±95.6)	4.8	1.9
General anesthesia	53721 (99.5)	2773 (99.5)	30680 (99.6)	1.1	2
Total intravenous anesthesia	10676 (19.8)	577 (20.7)	5793 (18.8)	3.2	0.8
RBC transfusion	8278 (15.3)	458 (16.4)	5530 (18.0)	4.7	4.7
Continuous infusion of inotropes	37279 (69.1)	1940 (69.6)	21191 (68.8)	1.2	1.2

Data are presented as n (%) or mean (±standard deviation).

IPW, inverse probability weighting; ASD, absolute standardized mean difference; RBC, red blood cell.

ASD was defined as absolute difference in means divided by pooled standard deviation between the three groups and ASD less than 0.1 was deemed to suggest a successful balance between the three groups.

### Clinical outcomes

**Fig 1:**
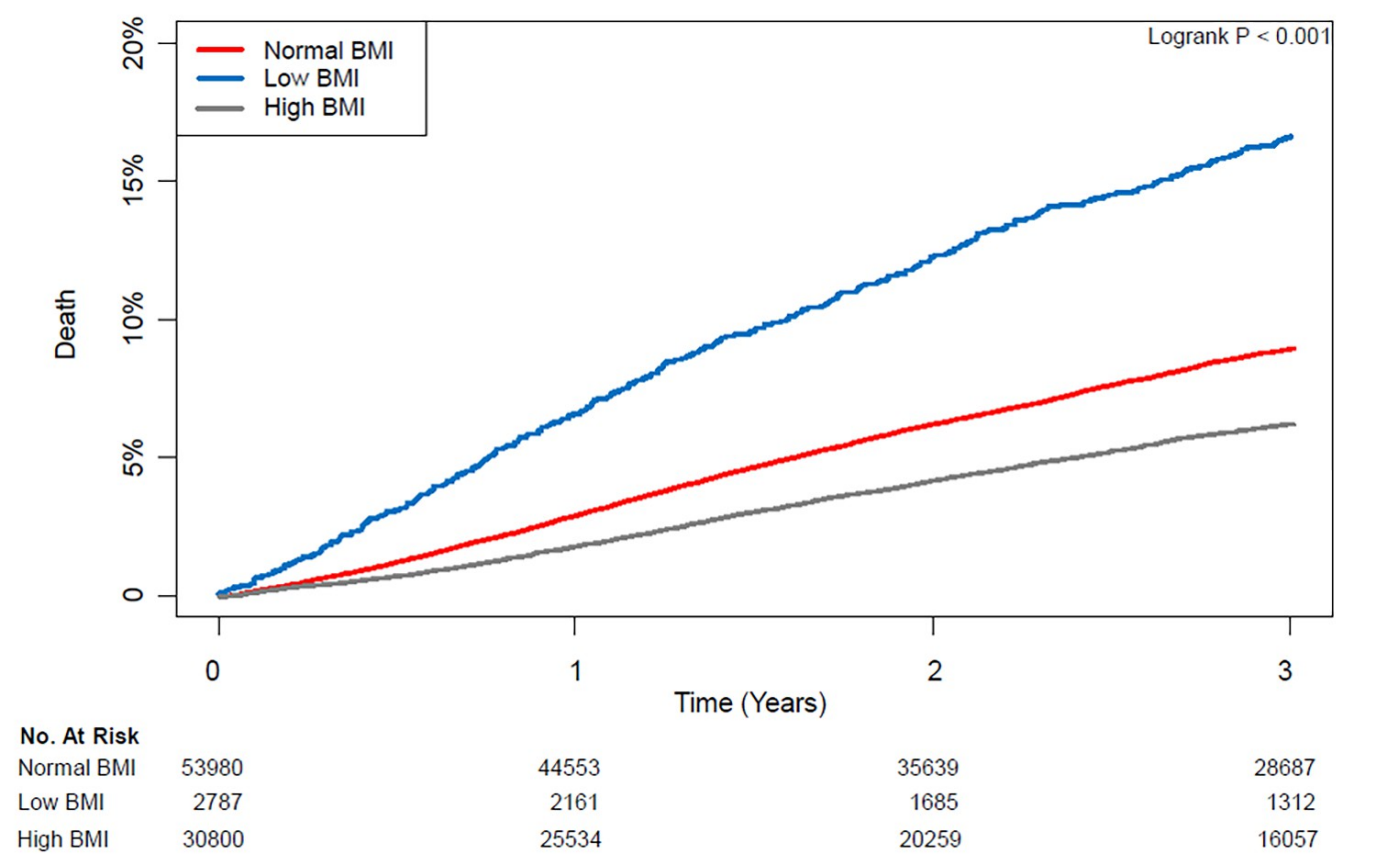
Kaplan–Meier curves of mortality according to body mass index during three years after cancer surgery.

Mortality in the three years after surgery was 6.4% (5,620/87,567) in the entire population, and the median follow-up durations for three-year mortalities was 818 days in the normal group, 765 days in the low BMI group, and 816 days in the high BMI group. Following IPW adjustment, three-year mortality of the high BMI group was lower than those of the normal (4.8% vs. 7.0%; HR: 0.69; CI: 0.64–0.74; p < 0.001) and low BMI (4.8% vs. 13.0%; HR: 0.38; CI: 0.35–0.42; p < 0.001) groups (Table 2). Mortality and recurrence rate during overall follow-up period were also lower in the high BMI group compared with the normal (6.9% vs. 9.4%; HR: 0.74; CI: 0.70–0.78; p < 0.001 for mortality and 5.3% vs. 6.5%; HR: 0.81; CI: 0.76–0.87; p < 0.001 for recurrence) and the low BMI (6.9% vs. 16.3%; HR: 0.44; CI: 0.41–0.48; p < 0.001 for mortality and 5.3% vs. 6.6%; HR: 0.84; CI: 0.74–095; p = 0.01 for recurrence) groups (Table 2). Survival curves are shown in Fig 1.

**Table 2:** Mortalities and recurrence rate according to preoperative body mass index.

	Normal (n *=* 53980)	Low BMI (n *=* 2787)	High BMI (n *=* 30800)	Low vs. High BMI
*Three-year mortality*, *No (%)*	3793 (7.0)	363 (13.0)	1464 (4.8)	
IPW adjusted HR (CI)		1.79 (1.64–1.96)	0.69 (0.64–0.74)	0.38 (0.35–0.42)
p-value		<0.001	<0.001	<0.001
*Five-year mortality*, *No (%)*	4687 (8.7)	433 (15.5)	192. (6.2)	
IPW adjusted HR (CI)		1.74 (1.60–1.89)	0.73 (0.69–0.78)	0.42 (0.39–0.46)
p-value		<0.001	<0.001	<0.001
*Overall mortality*, *No (%)*	5093 (9.4)	454 (16.3)	2113 (6.9)	
IPW adjusted HR (CI)		1.68 (1.55–1.82)	0.74 (0.70–0.78)	0.44 (0.41–0.48)
p-value		<0.001	<0.001	<0.001
*One-year mortality*, *No (%)*	1539 (2.9)	176 (6.3)	545 (1.8)	
IPW adjusted HR (CI)		1.96 (1.75–2.20)	0.63 (0.57–0.69)	0.32 (0.28–0.36)
p-value		<0.001	<0.001	<0.001
*30-day mortality*, *No (%)*	186 (0.3)	31 (1.1)	85 (0.3)	
IPW adjusted HR (CI)		2.26 (1.36–3.77)	0.80 (0.54–1.21)	0.36 (0.20–0.62)
p-value		0.001	0.2	<0.001
*Recurrence*, *No (%)*	3520 (6.5)	184 (6.6)	1645 (5.3)	
IPW adjusted HR (CI)		0.97 (0.86–1.09)	0.81 (0.76–0.87)	0.84 (0.74–0.95)
p-value		0.48	<0.001	0.01

IPW, inverse probability weighting; HR, hazard ratio; CI, confidence interval.

We maintained the alpha level at 0.05 for the primary outcome by adopting a post hoc Bonferroni correction, with a significance criterion of 0.0167 (0.05/3), and reported 98.3% confidence intervals (CIs).

**Fig 2:**
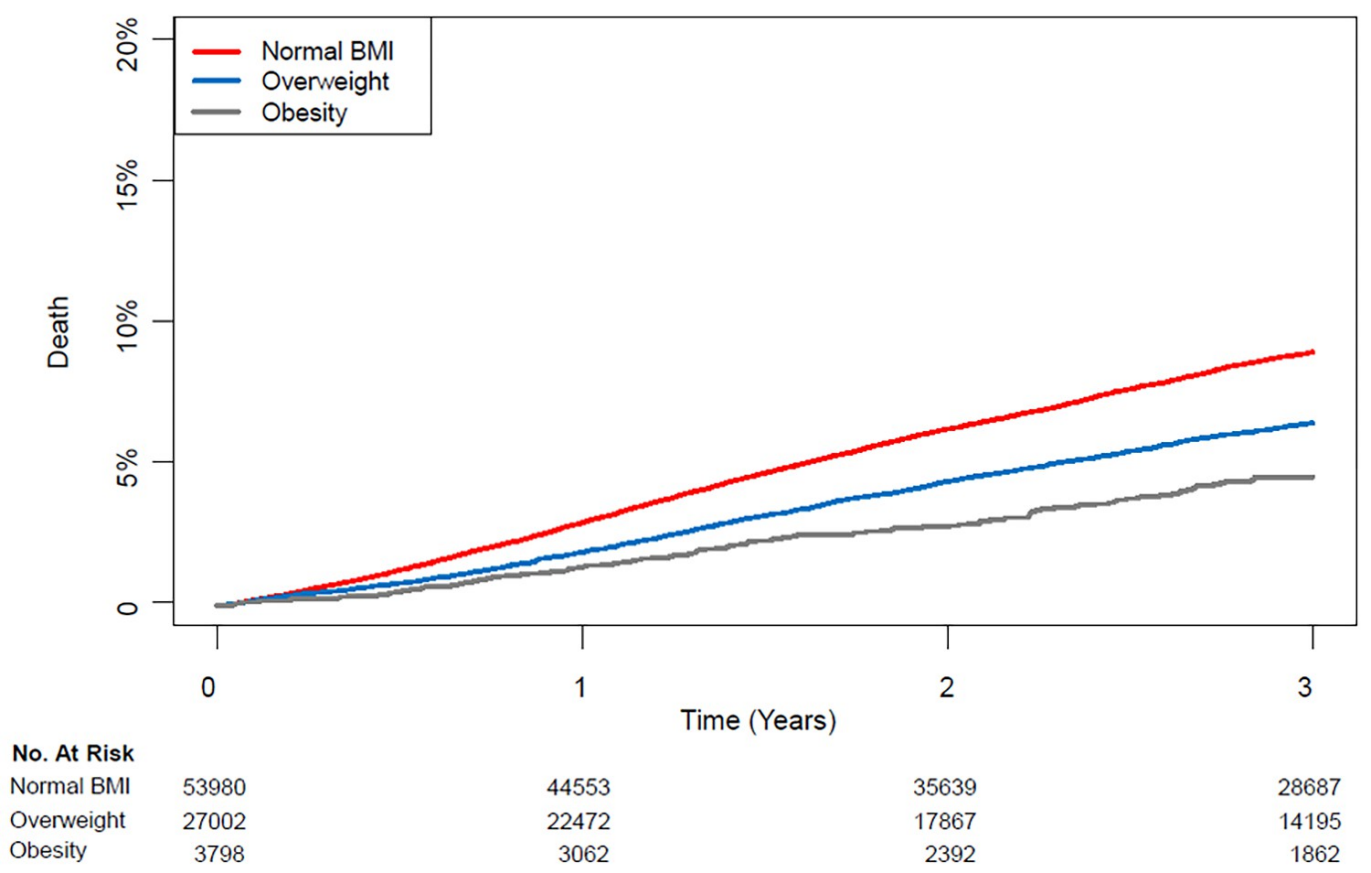
Kaplan–Meier curves of mortality of the normal, overweight, and obesity groups during three years after cancer surgery.

**Fig 3:**
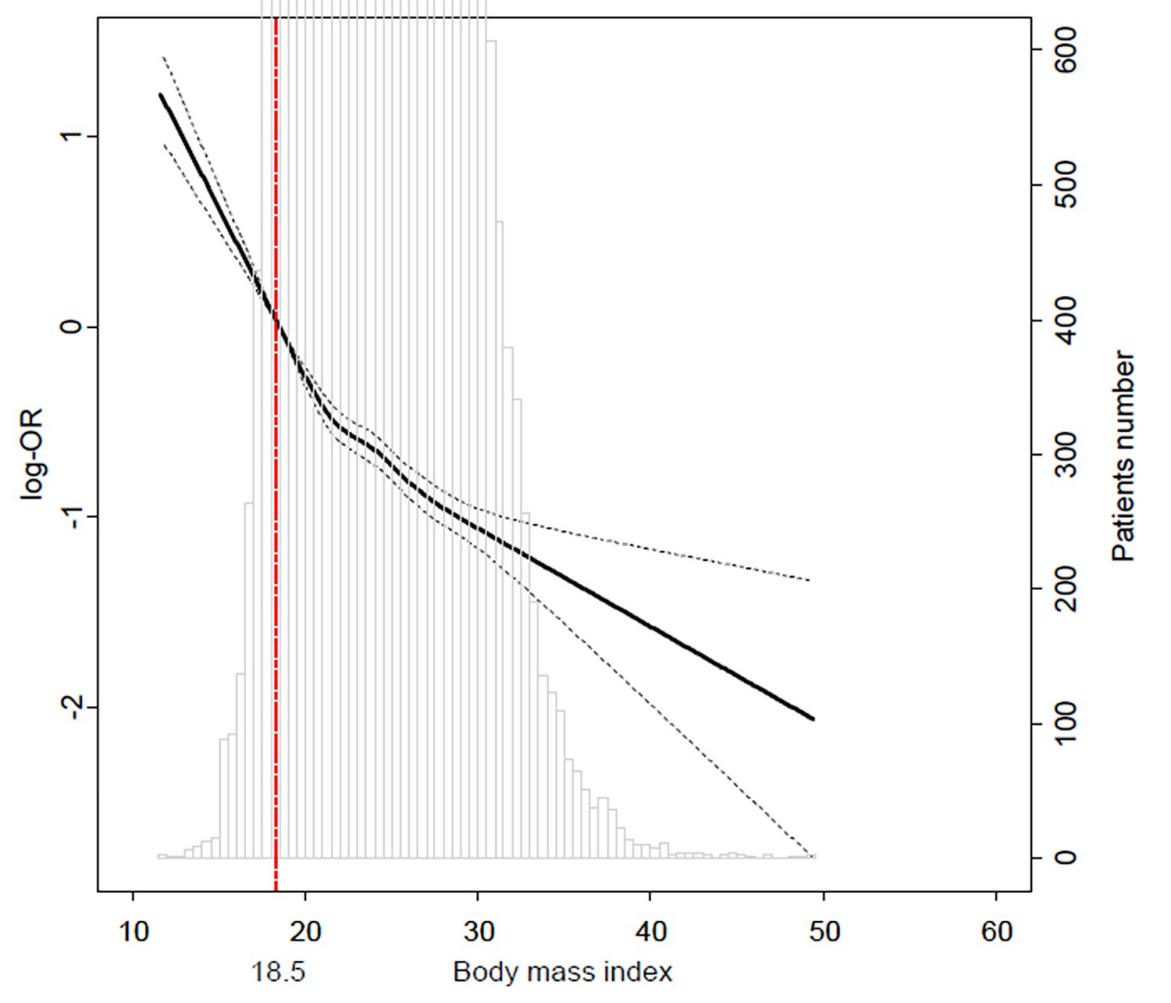
Smooth plot of HRs for mortality during one year according to body mass index.

The high BMI group was further divided into overweight and obese patients, defined as BMI equal to or greater than 30 kg/m^2^ (Supplemental Digital Content 1). The overweight and obesity groups were compared with the normal group in a pairwise fashion. After an adjustment using IPW, the three-year mortalities were significantly lower in the overweight (7.0% vs. 5.0%; HR: 0.72; CI: 0.67–0.77; p < 0.001) and obesity groups (7.0% vs. 3.3%; HR: 0.57; CI: 0.50–0.65; p < 0.001) groups compared with the normal group (Table 3). Comparison between the overweight and obese groups revealed that mortality was significantly lower for the obesity group (5.0% vs. 3.3%; HR: 0.79; CI: 0.69–0.91; p < 0.001) (Fig 2). The change in HR for three-year mortality according to preoperative BMI is shown in Fig 3.

**Table 3:** Mortalities and recurrence rate of the normal, overweight and obesity groups.

	Normal (n *=* 53980)	Overweight (n *=* 27002)	Obesity (n *=* 3798)	Overweight vs. Obesity
*Three-year mortality*, *No (%)*	3793 (7.0)	1338 (5.0)	126 (3.3)	
IPW adjusted HR (CI)		0.72 (0.67–0.77)	0.57 (0.50–0.65)	0.79 (0.69–0.91)
p-value		<0.001	<0.001	<0.001
*Five-year mortality*, *No (%)*	4687 (8.7)	1750 (6.5)	170 (4.5)	
IPW adjusted HR (CI)		0.75 (0.72–0.79)	0.57 (0.51–0.63)	0.75 (0.72–0.79)
p-value		<0.001	<0.001	<0.001
*Overall mortality*, *No (%)*	5093 (9.4)	1931 (7.2)	182 (4.8)	
IPW adjusted HR (CI)		0.77 (0.72–0.81)	0.61 (0.55–0.59)	0.80 (0.71–0.90)
p-value		<0.001	<0.001	<0.001
*One-year mortality*, *No (%)*	1539 (2.9)	496 (1.8)	49 (1.3)	
IPW adjusted HR (CI)		0.65 (0.60–0.72)	0.50 (0.41–0.60)	0.76 (0.62–0.93)
p-value		<0.001	<0.001	0.01
*30-day mortality*, *No (%)*	186 (0.3)	79 (0.3)	6 (0.2)	
IPW adjusted HR (CI)		0.83 (0.55–1.26)	0.83 (0.39–1.76)	1.00 (0.45–2.21)
p-value		0.29	0.56	0.99
*Recurrence*, *No (%)*	3520 (6.5)	1502 (5.6)	143 (3.8)	
IPW adjusted HR (CI)		0.84 (0.78–0.90)	0.62 (0.54–0.71)	0.73 (0.64–0.85)
p-value		<0.001	<0.001	<0.001

IPW, inverse probability weighting; HR, hazard ratio; CI, confidence interval.

We maintained the alpha level at 0.05 for the primary outcome by adopting a post hoc Bonferroni correction, with a significance criterion of 0.0167 (0.05/3), and reported 98.3% confidence intervals (CIs).

### Sensitivity analysis

**Fig 4:**
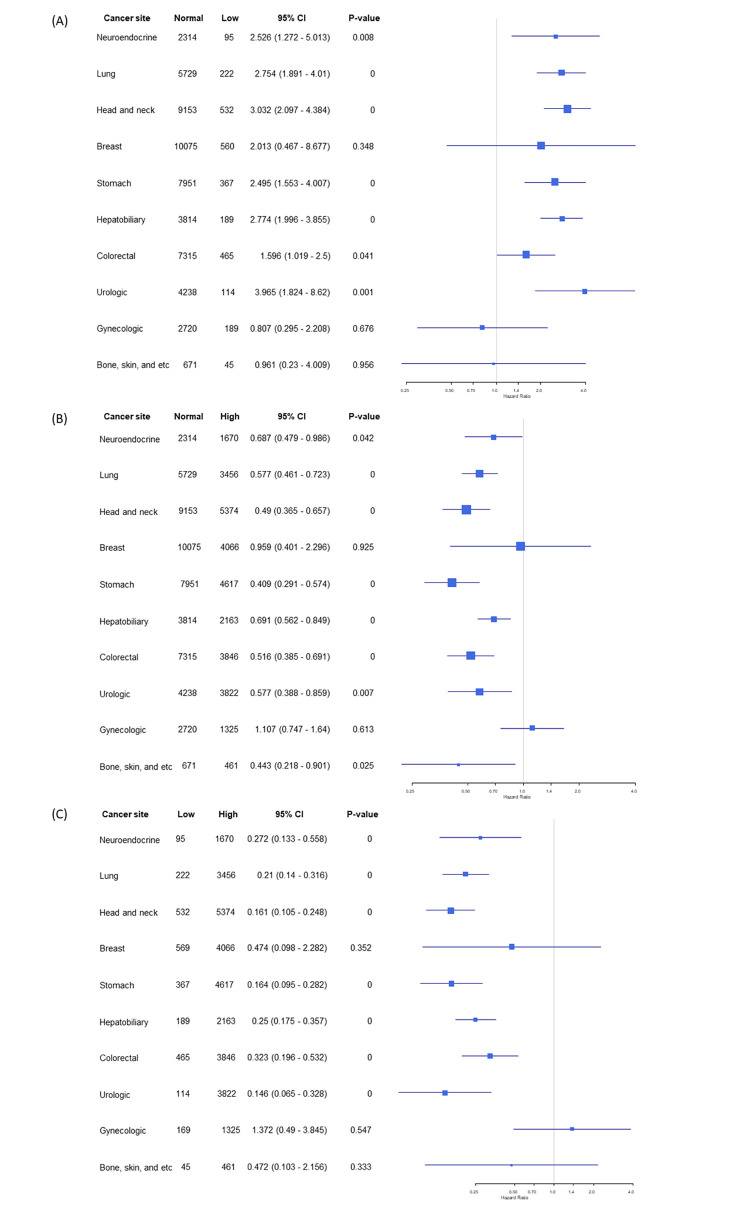
Subgroup analysis according to cancer sites in (a) normal vs. low BMI groups, (b) normal vs. high BMI groups, and (c) low vs. high BMI groups comparisons.

The observed association between three-year mortality and high BMI was significant in patients aged over 60 years old and hypertensive patients (HR: 0.60; CI: 0.54–0.68; p < 0.001 and HR: 0.66; CI: 0.59–0.75; p < 0.001) (Table 4). In subgroup analysis according to cancer site, three-year mortality was not significantly associated with high BMI in patients with breast and gynecological cancers (HR: 1.33; CI: 0.91–1.93; p = 0.14 for breast cancer and HR: 0.89; CI: 0.71–1.13; p = 0.35 for gynecological cancer) (Fig 4).

**Table 4:** Association between three-year mortality and preoperative body mass index in female and patients aged over 60 years old.

	Normal	Low BMI	High BMI	Low vs. High BMI
*Female*	n *=* 31263	n *=* 1829	n *=* 14447	
No (%)	496 (1.6)	50 (2.7)	195 (1.3)	
IPW adjusted HR (CI)	1 [reference]	1.52 (1.86–1.94)	0.90 (0.78–1.04)	0.59 (0.46–0.76)
p-value		0.001	0.14	<0.001
*Over 60 years old*	n *=* 20910	n *=* 1001	n *=* 13559	
No (%)	1027 (4.9)	122 (12.2)	374 (2.8)	
IPW adjusted HR (CI)	1 [reference]	2.15 (1.89–2.45)	0.60 (0.54–0.68)	0.28 (0.24–0.33)
p-value		<0.001	<0.001	<0.001
*No hypertension*	n *=* 40166	n = 2351	n = 17300	
No (%)	2366 (5.9)	261 (11.1)	653 (3.8)	
IPW adjusted HR (CI)	1 [reference]	1.91 (1.69–2.16)	0.66 (0.59–0.75)	0.35 (0.30–0.40)
p-value		<0.001	<0.001	<0.001
*No coronary artery disease*	n *=* 52493	n = 2730	n = 29489	
No (%)	3617 (6.9)	347 (12.7)	1348 (4.6)	
IPW adjusted HR (CI)	1 [reference]	2.68 (1.90–3.78)	0.87 (0.65–1.17)	0.33 (0.22–0.48)
p-value		<0.001	0.26	<0.001
*No metformin user*	n *=* 52435	n = 2742	n = 29575	
No (%)	3568 (6.8)	349 (12.7)	1360 (4.6)	
IPW adjusted HR (CI)	1 [reference]	1.11 (0.78–1.56)	0.92 (0.76–1.12)	0.83 (0.58–1.19)
p-value		0.48	0.31	0.22
*No statin user*	n *=* 50385	n = 2689	n = 27348	
No (%)	3442 (6.8)	336 (12.5)	1219 (4.5)	
IPW adjusted HR (CI)	1 [reference]	1.14 (0.81–1.61)	0.94 (0.77–1.13)	0.82 (0.22–0.48)
p-value		0.36	0.41	0.18
*No metastasis*	n *=* 52141	n = 2635	n = 30034	
No (%)	3008 (5.8)	291 (11.0)	1164 (3.9)	
IPW adjusted HR (CI)	1 [reference]	1.13 (0.79–1.60)	0.90 (0.74–1.10)	0.80 (0.55–1.16)
p-value		0.42	0.21	0.15

IPW, inverse probability weighting; HR, hazard ratio; CI, confidence interval.

## Discussion

This study compared mortality after cancer surgery by stratifying patients according to preoperative BMI and demonstrated that high BMI was associated with lower mortality during three years of follow-up. Furthermore, in the high BMI group, the mortality of obese patients was lower than that of the overweight and normal groups. This association was not significant in female patients and those undergoing surgery for breast and gynecological cancers.

Mortality of general population is lowest in those with a BMI within normal range^1^. Biologic evidence suggests that obesity may optimize the environment for tumor initiation, development, and progression by promoting and activating the mammalian target of rapamycin pathway [20]. This pathway plays a pivotal role in controlling cell growth, proliferation, and regulating essential metabolic processes, through reactive oxygen species [20]. The International Agency for Research on Cancer indicated that excessive body fatness is a risk factor of most cancers [4]. However, epidemiologic data have demonstrated that a high BMI may have a paradoxical protective effect against mortality due to cancer [21], although it has not been as well demonstrated in oncologic patients as it has been in cardiovascular disease or chronic pulmonary and renal disease patients [9–11,22].

This study showed that high BMI was associated with lower mortality after cancer surgery. Our finding is consistent with previously reported results from surgical patients [9,10]. Obesity paradox in surgical patients can be explained by various cytokines released by adipose tissue that may play key roles in protective effect against inflammation by regulating endovascular homeostasis and neutralizing tumor necrosis factor-α [9,10]. Additionally, obesity may protect patients from the adverse effects of malnutrition and energy expenditures related to their surgical procedures [9]. The physiologic reserve offered by excess adipose tissue could be even more important in cancer patients than those with other diseases [23]. In patients undergoing cancer surgery, the obesity paradox has previously been reported for gastrointestinal [14,15], lung [16], and renal cancer surgeries [17]. In this study, the protective effect of high BMI on mortality was observed for a three-year follow-up rather than short period of follow-up. This finding suggests that the beneficial effects of high BMI in cancer surgery may extend beyond providing a physiologic reserve or regulating inflammation during the immediate postoperative period.

Recently an additional explanation was proposed that a high BMI may be associated with lower stage, smaller tumor size, and less aggressive biological subtypes of cancer. In renal cell carcinoma, it was shown that obese patients were less likely to present with an advanced stage owing to downregulation of fatty acid synthase [17]. We found a significantly lower rate of recurrence in the high BMI group, suggesting that the disease state of cancer may be related to the observed association between BMI and mortality. Lastly, increased awareness during surgical procedures or diagnosis at an earlier stage of cancer due to more frequent medical check-ups in obese patients may also contribute to the lower mortality observed in the high BMI group [24].

Complex biologic mechanisms underlie the association between obesity and cancer. In our sensitivity analysis, potential confounders showed an association with the observed association. High BMI was not associated with mortality in female patients and those undergoing surgeries for breast and gynecological cancers which were also female dominant. The association between BMI and cancer was previously shown to differ between sexes in a large prospective study of more than 900,000 adults, and cancer types that are highly associated with BMI were distinct between sexes [25]. In previous studies, breast and gynecological cancers were included as cancers for which the risks are markedly increased by high BMI [5,25]. The risk of developing breast and gynecological cancers, which are hormone receptor-positive diseases, was increased in post-menopausal women by high BMI, but decreased in pre-menopausal women by high BMI [5]. In addition, previous studies reported that the obesity paradox was observed only in older patients [26,27], so we conducted sensitivity analysis in patient older than 60 years. Our result showed that high BMI was associated with decreased mortality after cancer surgery regardless of age. We also conducted subgroup analysis on metabolic drugs that are known for antitumor effect [28]. And the use of metabolic drug may also play a role in this association. Considering that our study enrolled cancer patients undergoing various types of cancer surgery, the role of potential confounding variable needs further investigations.

Lastly, the feasibility of using BMI as a measure of obesity needs to be discussed. As the most commonly used scale to measure obesity, the efficacy and cut-off value of BMI to predict cardiovascular events are relatively well established [29]. However, limitations persist in its use for cancer patients [30]. The major limitation is that BMI does not reflect body composition which has shown to be closely related to outcomes in oncology [31]. In addition, variation in BMI according to age or ethnicity has not been widely investigated [24]. Indeed, the cut-off value that we used to define our high BMI group is used to define obesity in the 2018 Korean Society for the Study of Obesity Guidelines [32], but only overweight in the CDC guideline. Therefore, we further divided the high BMI group into the overweight and obesity subgroups and compared the outcomes of these groups with the normal group. Our results showed that patients in the obesity group showed lower mortality than those in the overweight group, and we again found an inverse relation between risk of mortality and BMI. Despite these limitations, BMI is a simple convenient, and widely used measurement of obesity, and the clinical implication of our findings are that BMI could be considered when predicting outcomes of cancer surgery. More careful postoperative monitoring may be needed for those with low BMI, but the clinical efficacy of weight control in patients scheduled for cancer surgery remains unclear and requires further investigation.

The following limitations should be considered when interpreting the results of this study. First, as a single-center, observational study, the results might have been affected by confounding factors; even a rigorous statistical adjustment could not correct for unmeasured variables. Because all types of various cancer surgery were included in this study, the stage of cancer could not be considered in the analysis. In addition, the mortality can be largely differed by subtypes of cancer at the same site, but this could also not be retained in the analysis. Second, BMI may not adequately reflect body fat mass, and we were not able to assess the relation between morbid obesity and mortality, because of the low incidence of morbidly obese patients in our study. Third, ethnic difference should be considered, since our cohort consisted of mostly Asian population. The diagnostic criteria for obesity and the suggested cut-off values of BMI for defining obesity differ by ethnicity. As our study population was Asian, our results may not be applicable to patients in Western countries. Despite these limitations, we showed that high BMI was associated with reduced risk for mortality using a cohort including all types of cancer surgery. The results of this study may affect future clinical investigations and daily practices.

In conclusion, in patients undergoing cancer surgery, high BMI appeared to be associated with lower mortality during three years of follow-up. But, considering this association did not appear in female, patients with breast or gynecological cancer, and patients without using metabolic drugs, further investigations should be needed in the future.
